# From nutritional optimization to consumer acceptance: sensory and nutritional evaluation of culturally adapted recipes for type 2 diabetes in Benin

**DOI:** 10.3389/fnut.2026.1845418

**Published:** 2026-06-26

**Authors:** Halimatou Alaofè, Bissola Malikath Bankolé, John Ehiri, Waliou Amoussa Hounkpatin

**Affiliations:** 1 Department of Health Promotion Sciences, Mel and Enid Zuckerman College of Public Health, University of Arizona, Tucson, AZ, United States; 2 School of Nutrition and Food Science and Technology, Faculty of Agricultural Sciences of the University of Abomey-Calavi (FSA-UAC) Campus d' Abomey-Calavi, Calavi, Benin

**Keywords:** cultural adaptation, linear goal programming, medical nutrition therapy, sensory evaluation, sub-Saharan Africa, type 2 diabetes

## Abstract

**Background:**

Medical nutrition therapy (MNT) is central to type 2 diabetes (T2D) management; however, adherence remains challenging in sub-Saharan Africa due to economic constraints, food insecurity, and limited cultural adaptation of dietary recommendations. Few studies have integrated dietary optimization, cultural adaptation, sensory evaluation, and laboratory nutrient analysis within a single translational framework. This study aimed to develop and evaluate culturally adapted, nutritionally optimized Beninese recipes for adults with T2D by assessing sensory acceptability and nutritional composition.

**Methods:**

A mixed-method exploratory study was conducted using 14 culturally adapted recipes developed through linear goal programming (LGP), expert validation, and cultural adaptation procedures. Consumer sensory evaluation was conducted among 30 adults with and without T2D using a 5-point hedonic scale assessing taste, aroma, texture, color, and overall liking. Friedman and Mann–Whitney U tests were used for statistical analyses. Laboratory nutrient analyses were performed using standard AOAC methods, and nutrient composition was reported per 100 g as consumed and per individual serving.

**Results:**

Overall sensory acceptability was high across recipes, with overall liking scores ranging from 4.0 ± 0.6 to 4.8 ± 0.5. Friedman tests showed no significant differences in sensory ratings across recipes (all *p* > 0.05). Sensory ratings were generally similar between participants with and without T2D, with only a small difference observed for aroma ratings (*p* = 0.034, r = 0.107). Per-serving energy values ranged from 204.3 ± 24.7 kcal to 552.4 ± 15.3 kcal. All recipes met the predefined acceptability threshold (overall liking ≥ 4.0), and participant feedback identified only minor refinements related to seasoning or texture.

**Conclusion:**

Traditional Beninese recipes can be reformulated to align with T2D dietary recommendations without compromising sensory acceptability. By integrating dietary optimization, cultural adaptation, sensory evaluation, and laboratory nutrient analysis, this study provides a practical framework for culturally acceptable food-based diabetes interventions in sub-Saharan Africa.

## 1 Introduction

Type 2 diabetes (T2D) is a major and rapidly growing public health challenge globally, with a disproportionate increase observed in sub-Saharan Africa (SSA). Recent estimates indicate that approximately 24 million adults were living with diabetes in SSA in 2021, a figure projected to more than double to 55 million by 2045, representing one of the fastest growth rates worldwide (1–3). This epidemiological transition is driven by rapid urbanization, dietary shifts toward energy-dense and processed foods, reduced physical activity, and limited access to preventive and clinical care. The rising burden of T2D in SSA poses significant challenges for already constrained health systems and underscores the urgent need for scalable, contextually appropriate interventions.

Nutrition plays a central role in the prevention and management of T2D, with strong evidence demonstrating that dietary modification improves glycemic control and reduces cardiometabolic risk (4, 5). However, adherence to medical nutrition therapy (MNT) remains a major challenge in SSA due to economic constraints, food insecurity, cultural food preferences, and limited dietary literacy (6–9). These barriers highlight the need for interventions that extend beyond nutrient adequacy to incorporate cultural acceptability, affordability, and feasibility within local food environments. Emerging evidence suggests that culturally adapted dietary interventions, those that preserve familiar ingredients, preparation methods, and sensory characteristics, are more likely to promote long-term dietary adherence and sustainable behavioral change (10–12).

Linear programming and related optimization approaches have increasingly been used to design diets that satisfy nutritional requirements while accounting for cost, sustainability, and cultural preferences (13–15). These approaches have demonstrated effectiveness in generating nutritionally adequate and affordable dietary patterns across diverse populations, including low-resource settings (16). For example, optimization models have been applied to develop culturally acceptable diets in European populations and to formulate nutritionally adequate complementary foods in African contexts (13, 17). Recent reviews further emphasize the potential of optimization frameworks to balance health, sustainability, and cultural acceptability in diet design (14). Despite these advances, most optimization-based studies have primarily focused on nutrient composition and cost, with limited attention to whether optimized meal plans translate into culturally acceptable, real-world recipes.

Although dietary optimization provides a powerful tool for designing nutritionally adequate diets, nutritional adequacy alone does not ensure sustained adherence. Food choices and long-term dietary adherence are strongly influenced by sensory attributes such as taste, aroma, texture, familiarity, and perceived acceptability (18–20). While some studies have examined acceptability of individual food products or culturally adapted interventions, few have systematically integrated dietary optimization, cultural adaptation, laboratory nutrient analysis, and consumer sensory evaluation within a single framework. As a result, there remains a critical gap between optimized dietary models and their translation into culturally acceptable and feasible dietary practices, particularly in SSA.

To address this gap, the present study builds on prior optimization work by translating nutritionally optimized meal plans into culturally adapted recipes and evaluating both nutritional composition and sensory acceptability. By integrating linear goal programming (LGP), expert validation, laboratory nutrient analysis, and consumer sensory testing, this study provides a comprehensive framework for bridging the gap between dietary modeling and real-world implementation. Specifically, we evaluated 14 culturally adapted optimized recipes for adults with T2D in Benin to determine whether these recipes can simultaneously achieve nutritional adequacy and high sensory acceptability. Findings from this study are intended to inform the implementation of culturally tailored MNT strategies within community-based diabetes programs such as Objectif Santé Diabète Bénin (OSanDiaBé) and to contribute to the broader evidence base on sustainable dietary interventions in SSA.

## 2 Materials and methods

### 2.1 Study design and setting

This study employed a mixed-methods design integrating dietary optimization, cultural adaptation, expert validation, consumer sensory evaluation, and laboratory nutrient analysis. Mixed-method approaches are recommended for complex nutrition interventions to enhance contextual relevance and methodological rigor (21). The study was conducted in Cotonou, southern Benin, between January and April 2025 as part of the OSanDiaBé program (22).

**Figure 1:**
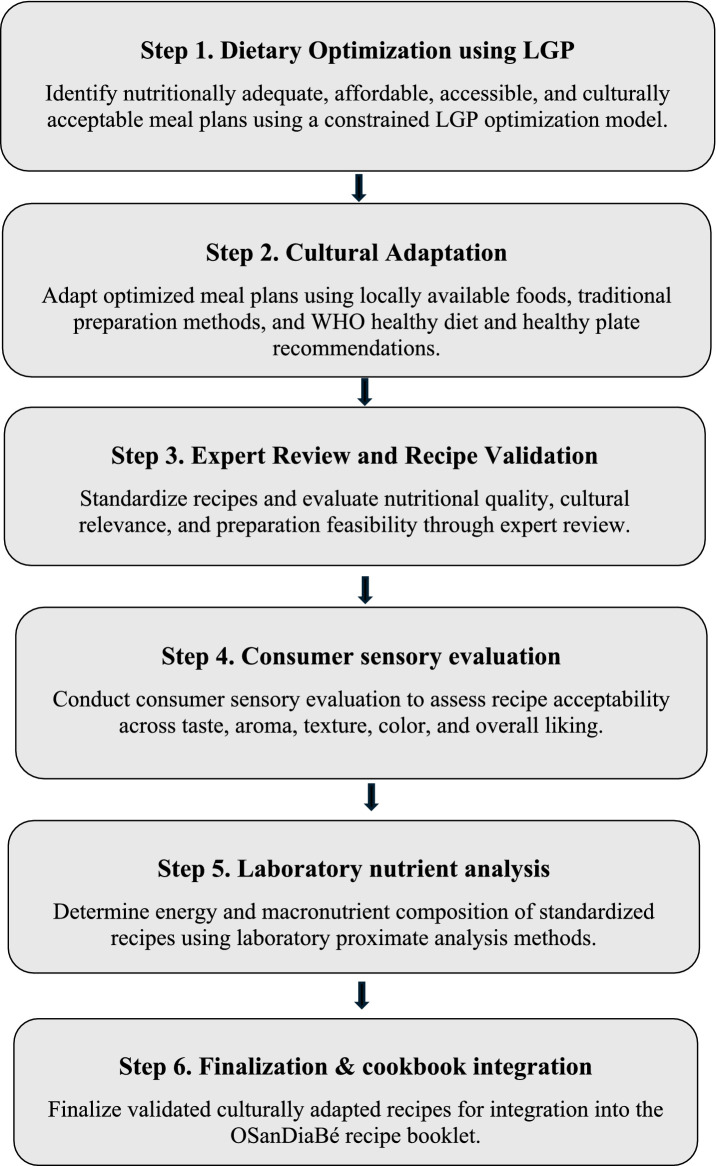
Six-step workflow for adapting and evaluating culturally appropriate recipes for individuals with type 2 diabetes (T2D), including nutritional optimization, expert validation, sensory testing, and nutrient analysis.

Ethical approval was obtained from the Institutional Review Board of the University of Arizona (IRB No: STUDY00002233) and the National Ethics Committee for Health Research of Benin (CNERS No: IRB00006860). Written informed consent was obtained from all participants prior to data collection. The methodological workflow included dietary optimization, cultural adaptation, expert review and recipe validation, consumer sensory evaluation, and laboratory nutrient analysis, as summarized in Figure 1.

### 2.2 Dietary optimization using linear goal programming (LGP)

Fourteen recipes were developed using LGP, a mathematical optimization technique widely used to design nutritionally adequate diets under multiple constraints (13–16). LGP enables simultaneous optimization of several objectives while accounting for real-world dietary constraints such as nutrient adequacy, affordability, portion size, and cultural acceptability for adults with T2D in Benin. Details of the optimization framework have been previously described by Alaofè et al. (23).

In summary, the model development followed a structured process including: (1) specification of nutrient targets based on World Health Organization (WHO) dietary recommendations for adults and individuals with T2D (24–28); (2) compilation of locally available foods with corresponding nutrient composition and cost data; and (3) definition of portion size and meal structure constraints consistent with local dietary practices and eating patterns.

The LGP model minimized deviations from predefined nutrient targets through a weighted objective function. Decision variables represented the quantities (g) of individual food items included in each optimized meal. The optimization objective aimed to minimize overall deviation from nutrient recommendations while maintaining realistic and culturally acceptable meal patterns. Constraints included: (i) nutrient requirements (energy, carbohydrates, protein, fat, fiber, and selected micronutrients), (ii) portion size limits and meal composition constraints, (iii) food cost considerations, and (iv) contextual factors reflecting the four pillars of food security: adequacy, availability, accessibility, and acceptability.

Optimization was performed iteratively using Python (Python Software Foundation, Wilmington, DE, USA) with the Gurobi optimization solver (Gurobi Optimization LLC, Beaverton, OR, USA). Iterative solution procedures were used to evaluate feasibility, reduce unrealistic food combinations, and improve cultural acceptability of optimized meal plans. Final optimized dietary outputs were subsequently translated into culturally adapted recipes for expert review, laboratory nutrient analysis, and consumer sensory evaluation, thereby linking mathematical optimization with real-world implementation.

### 2.3 Cultural adaptation

Optimized recipes were adapted from traditional Beninese dishes to preserve familiar ingredients, preparation methods, textures, and sensory profiles. For example, staple foods such as pâte (maize- or cassava-based dough) were reformulated through portion control and paired with larger servings of vegetable-based sauces rich in leafy greens (e.g., gboman, amaranth) and okra. Deep-fried preparations were modified by steaming or light sautéing, and palm oil quantities were reduced or partially replaced with controlled portions of vegetable oil. Refined grains were substituted with whole maize or mixed-grain options when feasible, and legumes such as cowpeas and lentils were incorporated into stews to enhance protein and fiber content. These modifications reduced added fats and refined carbohydrates while increasing dietary fiber and nutrient density.

Adaptations were guided by diabetes nutrition therapy principles (26–28) and WHO healthy diet recommendations (24, 25). This culturally grounded strategy aligns with evidence suggesting that culturally adapted interventions improve dietary adherence and sustainability in African contexts (10–12, 29).

### 2.4 Expert review and recipe validation

Recipe preparation and expert evaluation were conducted at the Laboratory of Food Sciences (LSA), Department of Nutrition, Food Science and Technology, University of Abomey-Calavi. Six nutrition professionals with expertise in dietetics, culinary science, and diabetes management participated in the review process.

Each recipe was prepared independently in triplicate by three trained graduate researchers using standardized household measures. Two structured consensus sessions were conducted to review ingredient quantities, preparation procedures, nutrient balance, sensory quality, and alignment with healthy plate recommendations.

Sensory attributes (appearance, color, aroma, texture, and taste) were evaluated using a standardized assessment form adapted from published sensory evaluation methodologies (19, 20, 30). Ratings were documented using structured evaluation forms accompanied by qualitative comments to guide recipe refinement. Final approval was granted only after consensus was reached on nutritional adequacy, sensory acceptability, and preparation feasibility. Finalized recipes, modifications, and rationale are presented in Table 1.

**Table 1:** Recipe modifications and nutritional rationale for T2D-adapted dishes*.

**No.**	**Original Recipe**	**Modifications and Rationale**
Breakfast		
1	Bread with egg, vegetable stew, and skimmed milk	Oil was substantially reduced (30 → 6 g) and salt reduced (10 → 2 g) to lower fat and sodium content. Bread and skimmed milk portions were maintained to preserve energy and lean protein balance.
2	Soy-enriched porridge with pâté (savory fried fritters)	Roasted soybean flour was reduced (200 → 150 g) and water increased (400 → 560 g) to improve texture and serving consistency. Milk was removed to lower total energy and fat content.
3	Oat porridge with bean fritters (Ata) and skimmed milk	Oats were reduced (200 → 100 g) and skimmed milk slightly reduced (150 → 125 g) to lower energy density and achieve an appropriate serving consistency.
4	Atassi (rice and beans dish) with vegetable stew, eggs, and apple	Rice and water quantities were increased (150 → 214 g rice; 600 → 1,320 g water) to improve cooking consistency. Chili pepper was reduced (~50%) to limit excessive spiciness, and bell pepper was added to increase vegetable variety and color. Eggs and cowpeas were retained to ensure adequate high-quality protein.
Lunch/Dinner		
5	White rice with tomato sauce and grilled fish	Water was increased (600 → 700 g) to improve sauce texture. Oil was reduced (30 → 12 g) and salt reduced (10 → 8 g) to lower total fat and sodium. Grilled fish was retained to maintain adequate lean protein while controlling portion size.
6	Riz au gras (tomato-based rice dish) with vegetable stew and grilled chicken	Palm oil was replaced with unsaturated vegetable oil and reduced (30 → 15 g) to lower energy density. Chili pepper was reduced (15 → 10 g). Tomato (150 → 306 g) and onion (100 → 128 g) were increased to improve micronutrient density. Water was reduced to achieve a firmer texture.
7	Macaroni served with vegetable stew and grilled chicken	Fresh tomato was slightly increased (150 → 155 g) to enhance flavor and lycopene content. Oil was reduced (15 → 6 g) and salt reduced (10 → 5 g) to lower fat and sodium. Water was increased (500 → 630 g) to improve overall consistency.
8	Wassa Wassa (steamed cassava couscous) with vegetable stew and grilled fish	Cassava couscous, oil, tomato, onion, fish, and salt quantities were reduced to lower energy, fat, and sodium content. Chili pepper was slightly increased (5 → 10 g) to enhance flavor balance while maintaining reduced oil use.
9	Akassa (fermented maize dough) with vegetable sauce and smoked fish	Ingredient quantities were reduced to improve energy balance. Palm oil was substantially reduced (45 → 12 g), and salt was lowered. Amaranth, tomato, garlic, and ginger were retained to preserve micronutrient density and flavor quality.
10	Gambali (dehulled maize flour dough) with okra sauce and grilled fish	Gambali flour was reduced by 50% (300 → 150 g) to lower energy density. Vegetable (okra, tomato) and grilled fish portions were maintained to preserve fiber and lean protein balance for T2D dietary needs.
11	Telibô (yam flour dough) with tomato sauce, grilled fish, and crincrin (jute leaf sauce)	Yam flour was reduced (300 → 255 g) to improve texture and portion balance. Grilled fish and jute leaf sauce were maintained to preserve protein and fiber contributions and ensure appropriate sauce consistency.
12	Boiled yam with vegetable stew and grilled chicken	Ingredient portions were reduced to improve energy balance and portion control while maintaining vegetable and lean protein components.
13	Beans with fried tomato and grilled mutton	Vegetable oil, chili powder, garlic, ginger, and bread portions were reduced to lower fat and energy content. Water was increased to improve texture while limiting added oil.
14	Vegetable salad with chicken eggs	Recipe maintained without modification due to appropriate nutrient balance and low energy density.

*All modifications were guided by dietary recommendations for individuals with T2D, emphasizing low glycemic index, reduced fat and sodium, and improved nutrient density. Adjustments prioritized cultural acceptability, culinary feasibility, and affordability.

### 2.5 Consumer sensory evaluation

A mixed-method design combining quantitative hedonic scoring and qualitative feedback was employed to assess consumer acceptance (21, 31, 32).

#### 2.5.1 Participants

Thirty adults (*n* = 30), including 14 individuals with T2D and 16 without T2D, were recruited through community networks and the preliminary project phase. This sample size aligns with established sensory evaluation practices, where 25–50 participants are considered adequate for consumer hedonic testing (19, 20). Inclusion criteria included age ≥18 years, regular consumption of traditional foods, and willingness to attend all sessions. Exclusion criteria included food allergies to recipe ingredients and sensory impairments affecting taste perception.

#### 2.5.2 Sensory procedures

Recipes were prepared on the day of testing using standardized methods. Participants were briefed on study objectives, procedures, and scoring criteria by a trained moderator. Each recipe was evaluated using a 5-point hedonic scale (1 = dislike extremely; 5 = like extremely) across five attributes: taste, aroma, texture, color, and overall liking (19, 20). The 5-point scale was selected to facilitate comprehension in a mixed-literacy population and has been used in applied community-based sensory research (19, 20).

Each participant evaluated multiple recipes presented in randomized order using coded three-digit identifiers to minimize bias and order effects. Water was provided for palate cleansing between samples. Portion satisfaction was also recorded as yes/no.

#### 2.5.3 Qualitative feedback

Participants provided open-ended feedback regarding sensory attributes, portion size, preparation characteristics, and suggested recipe improvements. Integrating qualitative feedback with quantitative sensory ratings strengthened triangulation and enhanced the reliability and contextual interpretation of findings in applied sensory research (32).

Qualitative data were analyzed using thematic analysis following the framework proposed by Braun and Clarke (33). The analytical process included: (1) data familiarization through repeated review of participant comments; (2) generation of initial codes; (3) grouping of related codes into categories; (4) inductive development of themes; and (5) review, refinement, and interpretation of final themes.

Coding was conducted independently by two researchers using a data-driven (inductive) approach. Discrepancies in coding or theme interpretation were resolved through discussion and consensus to improve analytical consistency and credibility. Final themes were organized according to major sensory and practical domains, including taste, aroma, texture/consistency, portion size, and preparation-related feedback.

### 2.6 Laboratory nutrient analysis

Nutrient composition analyses were conducted in collaboration with the Central Food Safety Laboratory (LCSSA) and the Laboratory of Food Sciences (LSA). Recipes were prepared in triplicate under standardized conditions and homogenized prior to analysis.

Moisture content was determined by oven drying at 105 °C (degrees Celsius) to constant weight as part of standard proximate composition analysis. Protein content was measured using the Kjeldahl method according to Association of Official Analytical Chemists (AOAC) standards (34). Fat content was determined using Soxhlet extraction methods (34), and ash content was assessed by dry ashing at 550 °C for 8 h (34). Carbohydrate content was calculated by difference according to Food and Agriculture Organization (FAO) procedures (35).

Energy values were calculated using Atwater conversion factors based on measured carbohydrate (35), protein, and fat contents according to the following equation:

$${}^{"}\text{Energy}\;{\left(\text{kcal}\right)}^{"}=(4\times {}^{"}{\text{Carbohydrate}}^{"})+(4\times {}^{"}{\text{Protein}}^{"})+(9\times {}^{"}{\mathrm{Fat}}^{"})$$

Quality assurance procedures included equipment calibration, standardized analytical protocols, repeat measurements, and use of reference standards to ensure accuracy and reproducibility.

Nutrient composition was expressed per 100 g as consumed (fresh weight basis) to facilitate comparability across recipes. In addition, nutrient values per individual serving were calculated to improve clinical interpretation and applicability for medical nutrition therapy in adults with T2D.

### 2.7 Statistical analysis

Quantitative data were analyzed using STATA version 19 (StataCorp, College Station, TX, USA). Descriptive statistics (means, standard deviations [SD], frequencies, and percentages) were calculated for demographic variables, sensory ratings, and nutrient composition. Normality was assessed using the Shapiro–Wilk test, a widely used method for evaluating distributional assumptions in small-to-moderate sample sizes (36).

Because each participant evaluated multiple recipes, within-subject comparisons of sensory scores were conducted using the Friedman test, a non-parametric repeated-measures analysis appropriate for ordinal sensory data and related samples (37, 38). Planned *post-hoc* pairwise comparisons using Wilcoxon signed-rank tests with Bonferroni correction were specified *a priori* and were to be conducted only if significant overall differences were identified (39).

Differences in sensory evaluation scores between participants with and without T2D were assessed using the Mann–Whitney U test, an appropriate non-parametric method for independent group comparisons involving ordinal or non-normally distributed data (38, 39). Effect sizes (r) were calculated to aid interpretation of the magnitude and practical relevance of observed differences (40). Statistical significance was set at *p* < 0.05.

Boxplots were generated to visualize the distribution, variability, and potential outliers of sensory scores across recipes (41).

### 2.8 Recipe finalization and selection criteria

Recipe finalization followed a multi-criteria decision framework incorporating: (1) sensory acceptability (mean overall liking score), (2) qualitative participant feedback, (3) nutritional adequacy (alignment with T2D dietary recommendations), and (4) cultural relevance and preparation feasibility (19, 20, 26, 32). This framework was used to ensure that finalized recipes were nutritionally appropriate, culturally acceptable, and feasible for community-based implementation.

A threshold of mean overall liking ≥ 4.0 was used to indicate high acceptability, corresponding to “like moderately” or higher on the 5-point hedonic scale and consistent with sensory evaluation standards and consumer acceptance research (19, 20, 30). Recipes meeting this threshold and requiring only minor refinements (e.g., seasoning or texture adjustments) were retained and standardized for final use.

Standardization procedures included verification of ingredient quantities, preparation methods, serving sizes, and recipe consistency to improve reproducibility and facilitate integration into the OSanDiaBé recipe booklet. Similar multi-criteria approaches integrating sensory evaluation, nutritional quality, cultural relevance, and consumer feedback have been recommended in food product development and culturally adapted nutrition interventions (31, 32). This process ensured that final recipes achieved both nutritional and sensory objectives while supporting real-world applicability and scalability.

## 3 Results

### 3.1 Socio-demographic characteristics of participants

As shown in Table 2, a total of 30 participants completed the sensory evaluation sessions, including 14 individuals with T2D (46.7%) and 16 without T2D (53.3%). Participants were predominantly female (56.7%). Age distribution was evenly divided between participants aged <40 years (50.0%) and ≥40 years (50.0%). Most participants were employed (86.7%), primarily in non-governmental (43.3%) or self-employed occupations (43.3%), while smaller proportions were government employees (10.0%) or retired (3.3%).

**Table 2:** Socio-demographic characteristics of study participants (*n* = 30).

Characteristic	*n*	%
Health status		
With Type 2 Diabetes	14	46.7
Without Type 2 Diabetes	16	53.3
Sex		
Female	17	56.7
Male	13	43.3
Age (years)		
20–39	15	50.0
≥ 40	15	50.0
Work status		
Government employee	3	10.0
Non-government employee	13	43.3
Self-employed	13	43.3
Retired	1	3.3

### 3.2 Sensory acceptability of adapted recipes

#### 3.2.1 Overall acceptability

Consumer sensory evaluation results are presented in Table 3. Overall acceptability of the adapted recipes was high, with mean overall liking scores ranging from 4.0 ± 0.6 to 4.8 ± 0.5 on the 5-point hedonic scale (1 = dislike very much; 5 = like very much). All recipes met or exceeded the predefined high-acceptability threshold (≥4.0), and standard deviations were generally below 0.8, indicating relatively consistent ratings across participants.

**Table 3:** Mean sensory evaluation scores of adapted recipes based on a 5-point hedonic scale (*n* = 30)*.

**N°**	**Recipe**	**Taste**	**Aroma**	**Texture**	**Color**	**Overall liking**
1	Bread with egg, vegetable stew, and skimmed milk	4.4 ± 0.6	4.5 ± 0.8	4.0 ± 0.7	4.8 ± 0.4	4.5 ± 0.5
2	Soy-enriched porridge with pâté (savory fried fritters)	4.6 ± 0.6	4.8 ± 0.5	4.5 ± 0.8	4.7 ± 0.6	4.7 ± 0.5
3	Oat porridge with bean fritters (Ata) and skimmed milk	3.6 ± 1.0	4.5 ± 0.9	4.2 ± 0.8	4.6 ± 0.6	4.0 ± 0.6
4	Atassi (rice and beans dish) with vegetable stew, eggs, and apple	3.8 ± 0.8	4.5 ± 0.7	4.7 ± 0.5	4.7 ± 0.5	4.0 ± 1.0
5	White rice with tomato sauce and grilled fish	4.6 ± 0.6	4.6 ± 0.6	4.4 ± 0.8	4.7 ± 0.7	4.6 ± 0.6
6	Riz au gras (tomato-based rice dish) with vegetable stew and grilled chicken	4.7 ± 0.7	4.7 ± 0.5	4.8 ± 0.5	4.7 ± 0.6	4.8 ± 0.4
7	Macaroni served with vegetable stew and grilled chicken	4.4 ± 0.7	4.6 ± 0.6	4.6 ± 0.6	4.6 ± 0.6	4.6 ± 0.6
8	Wassa Wassa (steamed cassava couscous) with vegetable stew and grilled fish	4.1 ± 0.8	4.6 ± 0.7	4.6 ± 0.6	4.7 ± 0.7	4.3 ± 0.7
9	Akassa (fermented maize dough) with vegetable sauce and smoked fish	4.6 ± 0.9	4.8 ± 0.6	4.8 ± 0.4	4.7 ± 0.5	4.8 ± 0.5
10	Gambali (dehulled maize flour dough) with okra sauce and grilled fish	4.2 ± 0.8	4.4 ± 0.9	4.4 ± 0.7	4.6 ± 0.7	4.3 ± 0.8
11	Telibô (yam flour dough) with tomato sauce, grilled fish, and crincrin (jute leaf sauce)	4.6 ± 0.6	4.7 ± 0.6	4.6 ± 0.7	4.7 ± 0.6	4.6 ± 0.6
12	Boiled yam with vegetable stew and grilled chicken	4.6 ± 0.6	4.8 ± 0.5	4.6 ± 0.6	4.8 ± 0.6	4.7 ± 0.6
13	Beans with fried tomato and grilled mutton	4.8 ± 0.4	4.7 ± 0.6	4.7 ± 0.5	4.7 ± 0.5	4.8 ± 0.4
14	Vegetable salad with chicken eggs	4.4 ± 0.7	4.6 ± 0.7	4.6 ± 0.7	4.8 ± 0.5	4.6 ± 0.7

*Values are mean ± standard deviation. Scores ≥ 4.0 were interpreted as high acceptability. Hedonic scale anchors: 1 = dislike very much; 5 = like very much. Ratings represent pooled responses from participants with and without T2D.

Mean scores for taste, aroma, texture, and color ranged from 3.6 to 4.8, with texture and color scores generally exceeding 4.0 across recipes. Recipes combining staple foods with vegetable-based sauces and lean protein sources demonstrated strong multi-attribute sensory performance. In particular, Akassa with vegetable sauce and smoked fish, Riz au gras with vegetable stew and grilled chicken, and beans with fried tomato and grilled mutton demonstrated among the highest overall liking and taste scores. In contrast, some cereal-based porridges demonstrated comparatively lower, though still acceptable, taste scores. For example, oat porridge with bean fritters (Ata) and skimmed milk received a mean taste score of 3.6 ± 1.0, consistent with participant comments regarding mild seasoning and thicker texture.

#### 3.2.2 Friedman test results

Friedman tests were conducted to assess within-subject differences in sensory ratings across recipes, accounting for the repeated-measures design (data not shown). No statistically significant differences were observed across recipes for taste (Kendall’s *W* = 0.304, *p* = 0.891), aroma (Kendall’s *W* = 0.271, *p* > 0.99), texture/consistency (Kendall’s *W* = 0.250, *p* > 0.99), color/appearance (Kendall’s *W* = 0.264, *p* = 0.999), or overall liking (Kendall’s *W* = 0.280, *p* = 0.990). Consequently, planned *post-hoc* pairwise comparisons using Wilcoxon signed-rank tests with Bonferroni correction were not performed.

#### 3.2.3 T2D versus non-T2D comparisons

Comparisons of sensory ratings between participants with and without T2D using the Mann–Whitney U test showed generally similar acceptability patterns across groups. No statistically significant differences were observed for taste (*p* = 0.069, *r* = 0.092), texture/consistency (*p* = 0.791, *r* = 0.013), color/appearance (*p* = 0.148, *r* = 0.073), or overall liking (*p* = 0.535, *r* = 0.031) (data not shown). Although aroma ratings differed significantly between groups (*p* = 0.034), the associated effect size was small (*r* = 0.107), indicating limited practical significance. Overall, effect sizes were small across sensory attributes, suggesting broadly comparable sensory perceptions between participants with and without T2D.

#### 3.2.4 Boxplots

**Figure 2:**
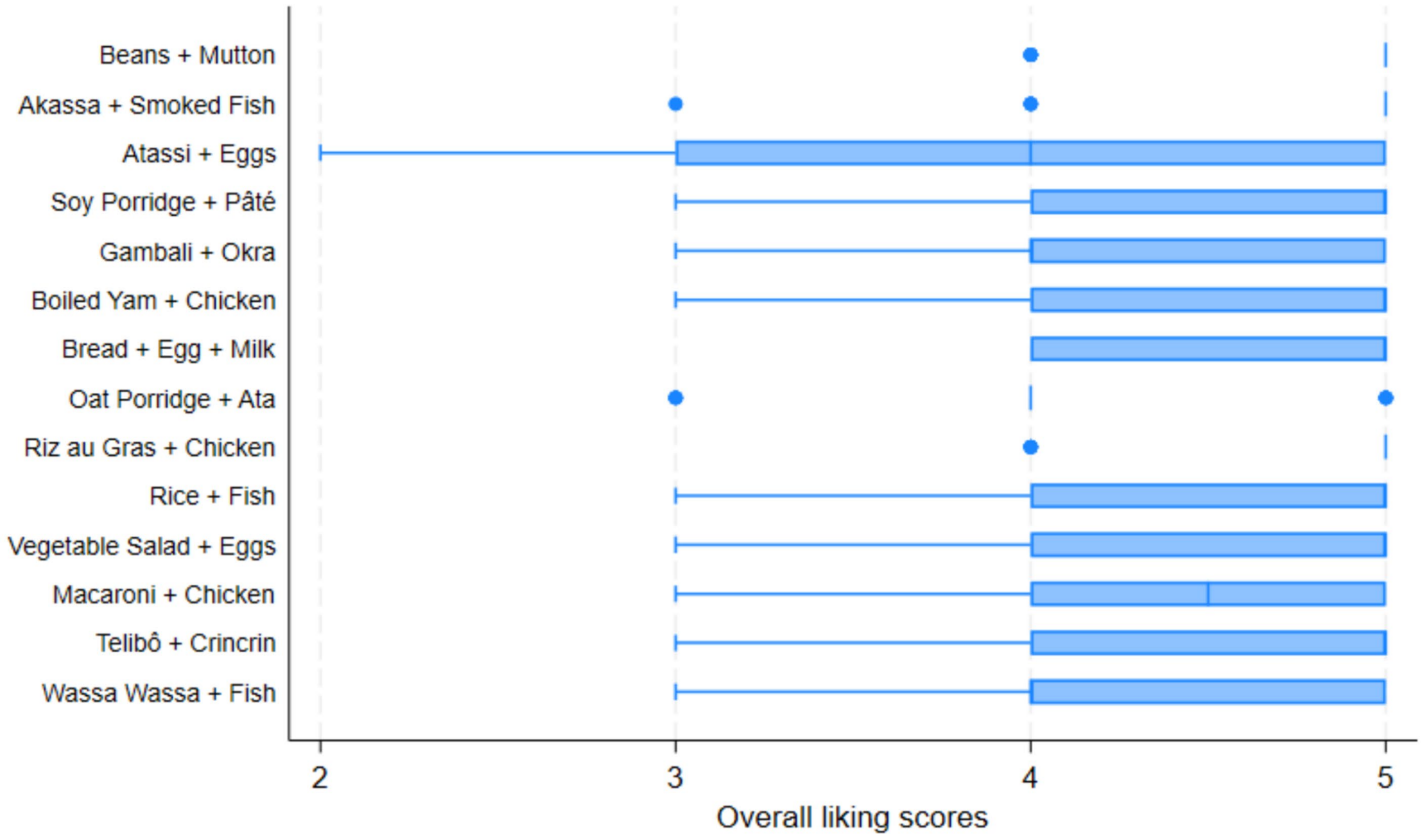
Distribution of overall liking scores across adapted recipes.

Boxplots illustrating the distribution and variability of overall liking scores across recipes are presented in Figure 2, while distributions for taste, aroma, texture/consistency, and color/appearance are shown in Supplementary Figure S1. Ratings clustered predominantly between scores of 4 and 5 across sensory attributes, indicating consistently favorable perceptions of the adapted recipes. Recipes such as Akassa with vegetable sauce and smoked fish (Recipe 2), Riz gras with grilled chicken (Recipe 9), Telibo with tomato and crincrin sauces and grilled fish (Recipe 13), and Wassa-wassa with grilled fish (Recipe 14) demonstrated relatively narrow interquartile ranges and median scores close to 5, reflecting high and consistent acceptability. In contrast, a small number of cereal-based recipes demonstrated greater variability in taste and aroma ratings, with occasionally lower outlier values. Nevertheless, median scores generally remained within the acceptable range. Overall, the boxplots supported the quantitative findings presented in Table 3 and confirmed generally high sensory acceptability across recipes.

#### 3.2.5 Qualitative sensory feedback

Qualitative feedback supported the quantitative findings and identified opportunities for minor refinements (Supplementary Table 1). Suggestions primarily related to seasoning intensity (e.g., salt, chili, garlic, ginger), sauce quantity, texture adjustments (e.g., porridge thickness, Akassa firmness), and portion size. No major acceptability concerns were reported. Recommendations were incremental and focused on flavor enhancement and presentation preferences. Overall, qualitative responses reinforced the high acceptability observed in hedonic ratings.

### 3.3 Energy and nutritional composition of the adapted recipes

Energy and macronutrient composition are presented in Table 4. Values are expressed per 100 g dry matter (DM) based on triplicate laboratory analyses.

**Table 4:** Energy and macronutrient composition of adapted recipes per individual serving (Mean ± SD).

***N***	**Recipes**	**Serving weight (g)**	**Energy (kcal/serving)**	**Carbohydrate (g/serving)**	**Protein (g/serving)**	**Fat (g/serving)**	**Fiber (g/serving)**
1	Bread with egg, vegetable stew, and skimmed milk	486	345.5 ± 24.9	37.4 ± 2.9	19.4 ± 0.5	13.1 ± 2.4	1.9 ± 0.5
2	Soy-enriched porridge with pâté (savory fried fritters)	309.1	247.3 ± 11.6	30.9 ± 2.5	11.4 ± 0.6	8.7 ± 0.6	1.5 ± 0.3
3	Oat porridge with bean fritters (Ata) and skimmed milk	407.2	288.7 ± 7.7	43.6 ± 1.6	9.4 ± 0.4	8.6 ± 0.4	2.0 ± 1.6
4	Atassi (rice and beans dish) with vegetable stew, eggs, and apple	451.67	405.1 ± 23.6	64.1 ± 5.0	15.8 ± 0.9	9.5 ± 1.4	5.9 ± 1.4
5	White rice with tomato sauce and grilled fish	404.9	311.4 ± 15.0	62.8 ± 3.2	10.5 ± 0.4	2.0 ± 0.8	3.6 ± 1.2
6	Riz au gras (tomato-based rice dish) with vegetable stew and grilled chicken	436.9	424.3 ± 27.5	67.7 ± 3.9	17.9 ± 0.4	10.5 ± 2.2	3.1 ± 1.3
7	Macaroni served with vegetable stew and grilled chicken	447.5	354.9 ± 27.1	59.1 ± 5.4	16.6 ± 0.9	5.8 ± 1.8	2.7 ± 1.3
8	Wassa Wassa (steamed cassava couscous) with vegetable stew and grilled fish	280.9	340.5 ± 12.2	54.8 ± 1.7	22.8 ± 0.3	3.4 ± 1.1	4.8 ± 0.8
9	Akassa (fermented maize dough) with vegetable sauce and smoked fish	564.2	552.4 ± 15.3	38.9 ± 2.8	29.3 ± 0.6	31.0 ± 1.1	3.9 ± 0.6
10	Gambali (dehulled maize flour dough) with okra sauce and grilled fish	413.5	204.3 ± 24.7	32.7 ± 5.4	12.8 ± 1.2	2.5 ± 1.2	2.1 ± 0.8
11	Telibô (yam flour dough) with tomato sauce, grilled fish, and crincrin (jute leaf sauce)	445.2	277.8 ± 11.5	50.3 ± 2.7	11.1 ± 0.4	3.6 ± 0.4	2.7 ± 0.4
12	Boiled yam with vegetable stew and grilled chicken	417.2	340.4 ± 33.7	48.4 ± 3.8	14.2 ± 0.8	10.0 ± 3.3	3.3 ± 0.0
13	Beans with fried tomato and grilled mutton	319	500.8 ± 39.5	57.4 ± 4.8	20.4 ± 0.6	21.1 ± 3.8	3.8 ± 0.3
14	Vegetable salad with chicken eggs	408.7	252.6 ± 12.9	22.1 ± 1.6	13.5 ± 0.4	12.3 ± 1.2	4.1 ± 0.8

Values are presented as mean ± standard deviation (SD). Nutrient composition is expressed per individual serving as consumed (fresh weight basis).

The energy and macronutrient composition of the adapted recipes are presented both per 100 g as consumed (Supplementary Table S2) and per individual serving (Table 4). Reporting nutrient composition per 100 g facilitated standardized comparison of nutrient density across recipes, whereas per-serving values provided clinically relevant interpretation for medical nutrition therapy in adults with T2D.

Per 100 g as consumed, energy density ranged from 49.8 ± 6.4 kcal for Gambali with okra sauce and grilled fish to 157.0 ± 14.7 kcal for beans with fried tomato and grilled mutton. Carbohydrate content ranged from 5.4 ± 0.4 g to 19.5 ± 0.6 g per 100 g, while protein content ranged from 2.3 ± 0.1 g to 8.1 ± 0.1 g. Fat content was highest in beans with fried tomato and grilled mutton and Akassa with vegetable sauce and smoked fish.

Per individual serving, energy values ranged from 204.3 ± 24.7 kcal for Gambali with okra sauce and grilled fish to 552.4 ± 15.3 kcal for Akassa with vegetable sauce and smoked fish. Carbohydrate content ranged from 22.1 ± 1.6 g in vegetable salad with chicken eggs to 67.7 ± 3.9 g in Riz au gras with vegetable stew and grilled chicken. Recipes incorporating legumes, fish, eggs, or meat generally demonstrated higher protein content, whereas recipes containing vegetables and legumes contributed relatively higher fiber values.

Overall, the adapted recipes reflected moderate carbohydrate distribution, inclusion of protein-rich foods, and incorporation of fiber-containing ingredients consistent with culturally tailored dietary approaches for T2D management.

### 3.4 Final recipe selection

Recipe finalization followed predefined multi-criteria selection criteria incorporating sensory acceptability, qualitative participant feedback, nutritional adequacy aligned with diabetes dietary recommendations, cultural relevance, and preparation feasibility. All 14 optimized recipes achieved the predefined acceptability threshold (mean overall liking score ≥ 4.0) and were retained for inclusion in the final recipe booklet.

Although some recipes demonstrated variability in specific sensory attributes, participant feedback and expert review indicated that only minor refinements, such as adjustments to seasoning, texture, or consistency, were needed. No recipe required exclusion or substantial reformulation following expert and consumer evaluation.

**Figure 3:**
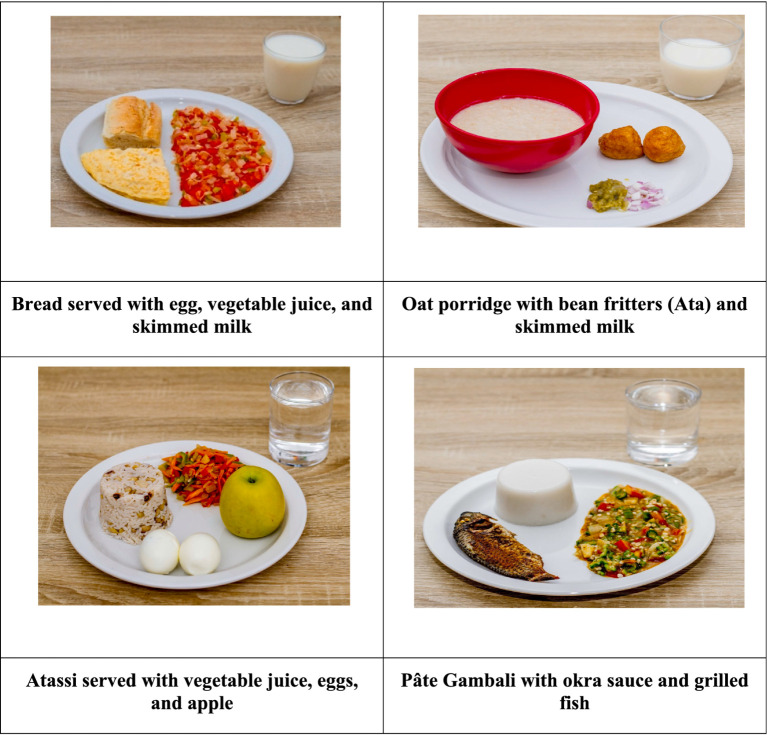
Examples of culturally adapted Beninese recipes developed for inclusion in the OSanDiaBé program.

Standardization procedures were subsequently completed to ensure reproducibility of ingredient quantities, preparation methods, and portion sizes across recipes. The finalized recipes constitute the basis of the OSanDiaBé recipe booklet developed to support culturally tailored medical nutrition therapy for adults with T2D in Benin. Representative examples of adapted recipes are presented in Figure 3.

## 4 Discussion

This study demonstrated that 14 culturally adapted and nutritionally optimized Beninese recipes achieved high sensory acceptability while aligning with dietary recommendations for T2D. Overall liking scores ranged from 4.0 to 4.8 on a 5-point hedonic scale, with consistently favorable ratings across taste, aroma, texture, and color. Friedman tests showed no significant differences in sensory ratings across recipes, suggesting broadly comparable acceptability among the adapted recipes. In addition, sensory perceptions were generally similar between participants with and without T2D, with only a small difference observed for aroma ratings. Nutrient analyses further demonstrated moderate carbohydrate distribution, inclusion of protein-rich foods, and incorporation of fiber-containing ingredients consistent with culturally tailored dietary approaches for T2D management (26–28).

These findings address an important gap between theoretical dietary optimization and real-world implementation. While previous optimization studies have focused primarily on nutrient adequacy, cost, or mathematical feasibility (13–17), relatively few have evaluated whether optimized meal plans can be translated into culturally acceptable recipes using consumer sensory evaluation and laboratory nutrient analysis within the same framework. The present study extends prior work by demonstrating that nutritionally optimized meal plans can be transformed into culturally grounded, food-based interventions suitable for adults with T2D in Benin (22, 23).

High sensory acceptability likely reflects the preservation of familiar ingredients, preparation methods, textures, and flavor profiles during recipe adaptation. Recipes incorporating traditional staples combined with vegetable-based sauces and lean protein sources consistently received favorable ratings, supporting evidence that familiarity and cultural relevance are major determinants of food choice and dietary adherence (18–20). Similar high acceptability has been reported in culturally adapted food-based interventions and optimized complementary food studies conducted in African settings (10–12, 17). Cultural familiarity has also been recognized as an important determinant of engagement and sustainability in public health nutrition interventions (29). Importantly, all recipes achieved the predefined acceptability threshold (overall liking ≥ 4.0), and qualitative feedback indicated that only minor refinements related to seasoning or texture were needed. Together, these findings suggest that improving nutritional quality does not necessarily compromise palatability when culturally meaningful culinary practices are preserved.

The absence of significant differences in overall liking between participants with and without T2D further supports the broader cultural appeal and feasibility of the adapted recipes. Although aroma ratings differed significantly between groups, the associated effect size was small, indicating limited practical significance. These findings are consistent with studies from SSA showing that culturally adapted dietary interventions are more likely to maintain acceptability and long-term adherence when traditional textures, spices, and cooking techniques are preserved (10–12, 42). Given the established relationship between sensory satisfaction and dietary adherence (7, 18–20), integrating sensory evaluation into dietary optimization frameworks may strengthen the sustainability of MNT interventions in low-resource settings. Sensory preferences and palatability are recognized determinants of food selection and long-term dietary adherence, particularly in chronic disease management (30, 31, 43, 44).

The nutritional findings also support the feasibility of aligning traditional Beninese recipes with evidence-based dietary recommendations for T2D management. Compared with many refined carbohydrate-dominant dietary patterns commonly reported in urbanizing African settings (8, 12), the adapted recipes incorporated more vegetables, legumes, and lean protein sources while controlling portion size and fat content. Per-serving analyses further improved clinical interpretability by demonstrating realistic meal-level nutrient profiles relevant for diabetes counseling and MNT implementation. Although dietary fiber values remained modest relative to WHO recommendations (24), incorporation of legumes, leafy vegetables, and whole-grain substitutions represented meaningful improvement over traditional formulations. Increased dietary fiber intake has been associated with improved glycemic control, satiety, and cardiometabolic outcomes among individuals with T2D (26–28, 45, 46).

This study also highlights the practical value of combining linear goal programming, cultural adaptation, sensory evaluation, and laboratory nutrient analysis within a single translational framework. Previous studies have shown that optimization approaches can generate nutritionally adequate and affordable dietary patterns (13–17); however, implementation challenges often arise when optimized meal plans fail to reflect local food practices and sensory preferences. By integrating community-informed adaptation and consumer testing, the present study translated dietary optimization into realistic food-based guidance that can be incorporated into community nutrition and diabetes programs such as OSanDiaBé (22, 23).

### 4.1 Translational and public health implications

From a public health perspective, culturally tailored food-based strategies may improve dietary adherence more effectively than generic dietary advice, particularly in settings where affordability, food availability, and cultural norms strongly influence eating behaviors (6–9). The recipes developed in this study relied primarily on affordable, locally available ingredients and standard household preparation methods, increasing feasibility for community and primary healthcare implementation. Embedding culturally familiar foods within diabetes education may also strengthen health literacy, self-efficacy, and long-term disease management (10, 11, 26).

Importantly, this study translated dietary optimization into actionable food-based guidance suitable for real-world implementation. Integration of culturally adapted recipes into programs such as OSanDiaBé may support community-based MNT delivery and contribute to more sustainable dietary behavior change among adults with T2D (22, 23). Similar translational approaches emphasizing implementation feasibility and contextual adaptation have been recommended for nutrition and chronic disease interventions in low- and middle-income countries (29, 47, 48).

### 4.2 Scalability and replicability

The six-step framework used in this study—dietary optimization, cultural adaptation, expert validation, consumer sensory evaluation, laboratory nutrient analysis, and standardized dissemination—provides a potentially scalable and replicable model for other low- and middle-income countries. Because the approach integrates scientific rigor with contextual relevance, it may support adaptation across diverse African food systems while maintaining core principles of nutritional adequacy, affordability, and cultural acceptability (13–17, 22, 23).

For broader implementation, the framework could be integrated into existing diabetes education and primary healthcare platforms through standardized recipe booklets, facilitator guides, and low-literacy educational materials. Adaptation to other settings may be achieved through contextual modification of recipes while preserving core nutritional and cultural principles.

### 4.3 Strengths and limitations

Several strengths should be noted. First, the study used a mixed-method design integrating quantitative sensory ratings, qualitative participant feedback, expert review, laboratory nutrient analysis, and dietary optimization using linear goal programming (LGP) (21, 31–34). Second, inclusion of participants with and without T2D enabled assessment of potential perceptual differences according to health status. Third, nutrient composition was reported both per 100 g as consumed and per individual serving, improving comparability, interpretability, and clinical relevance for MNT. Finally, the study adopted a translational framework linking dietary optimization, culturally adapted recipe development, consumer sensory evaluation, and practical implementation within a community-based diabetes nutrition context.

As an exploratory mixed-method study, the primary objective was to assess feasibility, sensory acceptability, and preliminary translational potential of culturally adapted optimized recipes for adults with T2D in Benin. Nevertheless, several limitations should be acknowledged. The modest sample size and single-site design limit generalizability beyond the study population. Although the sample size aligns with accepted sensory evaluation practices for exploratory consumer testing (19, 20), larger multi-site studies are needed to confirm acceptability across broader demographic and cultural groups. In addition, sensory evaluation reflects short-term acceptability rather than long-term dietary adherence or clinical effectiveness. Although recipes were developed in accordance with T2D dietary recommendations, glycemic index (GI) and glycemic load (GL) were not directly assessed. Future studies should incorporate glycemic response measurements and clinical outcomes to further evaluate the metabolic effects of culturally adapted optimized recipes. Furthermore, use of a 5-point hedonic scale, while appropriate for mixed-literacy populations, may have reduced sensitivity for detecting subtle differences in sensory perception compared with more detailed 7- or 9-point scales (19, 20, 30). Future research should evaluate the impact of culturally adapted optimized recipes on dietary adherence, glycemic control, weight management, and implementation outcomes in larger community-based trials (22, 23, 47, 48).

## 5 Conclusion

Traditional Beninese dishes can be reformulated to align with T2D dietary recommendations without compromising sensory acceptability. By integrating dietary optimization, cultural adaptation, consumer sensory evaluation, and laboratory nutrient analysis, this study provides a practical framework for translating optimized diets into culturally acceptable, real-world interventions in sub-Saharan Africa. Embedding culturally grounded, food-based strategies into programs such as OSanDiaBé may support sustainable improvements in dietary adherence and long-term diabetes management. This framework may also support the development of culturally acceptable and scalable food-based diabetes interventions across sub-Saharan Africa.

## Data Availability

The original contributions presented in the study are included in the article/Supplementary material, further inquiries can be directed to the corresponding author/s.
