# Supplementary figures and images for: From nutritional optimization to consumer acceptance: sensory and nutritional evaluation of culturally adapted recipes for type 2 diabetes in Benin
Source: Front Nutr. 2026 Jun 26;13:1845418. doi: 10.3389/fnut.2026.1845418 (PMC13371430; doi:10.3389/fnut.2026.1845418)

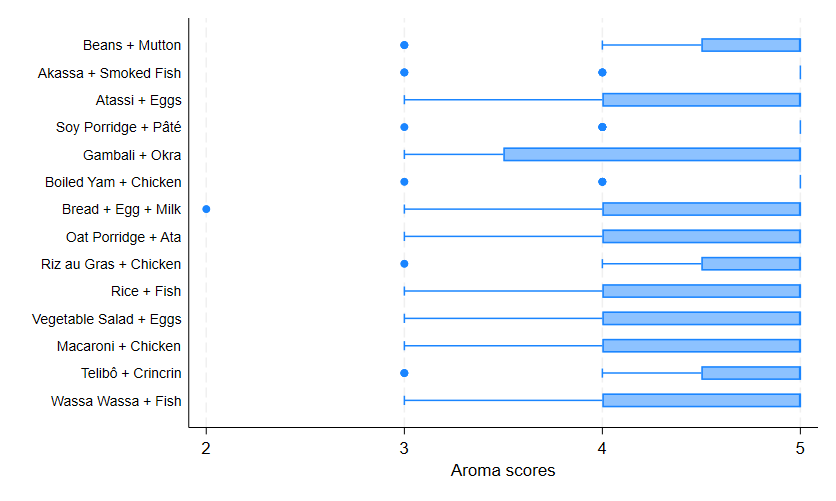
**Supplementary Figure S1.** Distribution of Sensory Attribute Scores Across Adapted Recipes


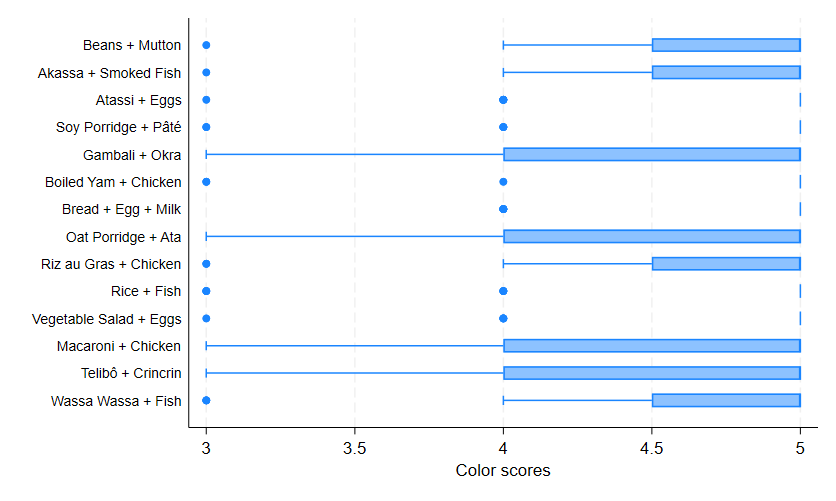


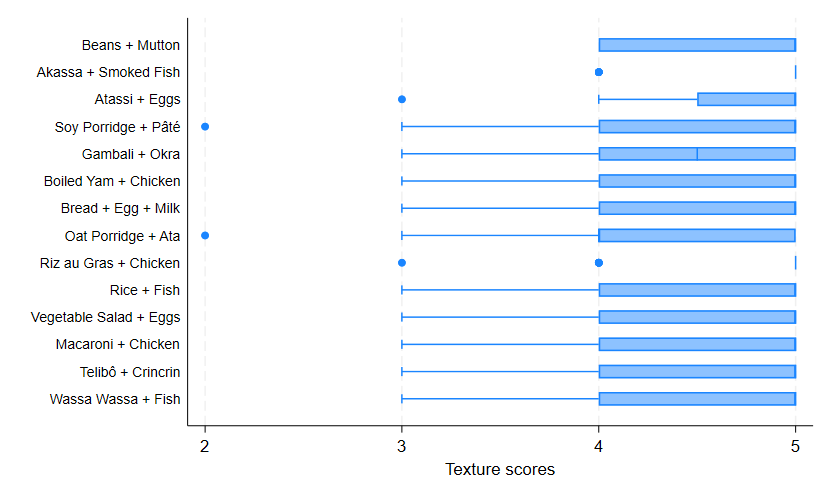

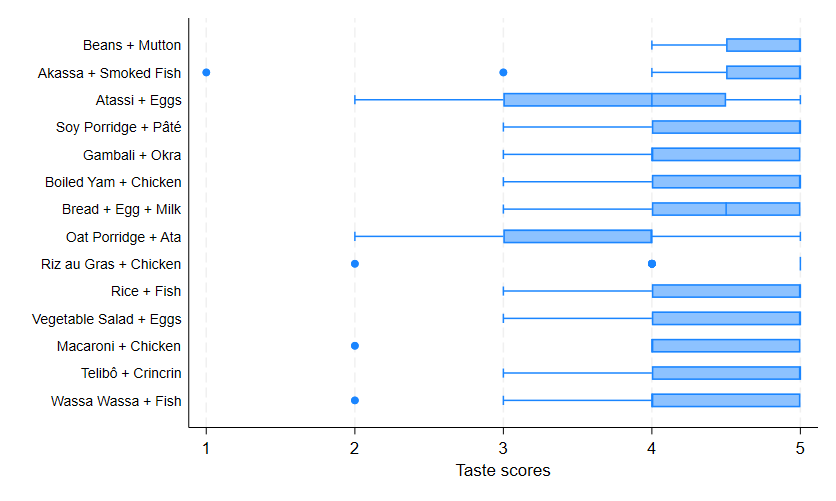

Supplement: Supplementary file 1 [file Supplementary_file_1.docx]
