# Supplementary material for: From nutritional optimization to consumer acceptance: sensory and nutritional evaluation of culturally adapted recipes for type 2 diabetes in Benin
Source: Front Nutr. 2026 Jun 26;13:1845418. doi: 10.3389/fnut.2026.1845418 (PMC13371430; doi:10.3389/fnut.2026.1845418)
Supplement: Supplementary file 2 [file Table_1.docx]

| **Supplementary Table S1.** Summary of participant qualitative sensory feedback and suggested recipe refinements grouped by sensory theme. | | | |
| --- | --- | --- | --- |
| **Recipe** | **Sensory attribute** | **Participant feedback (summary)** | **Suggested refinement** |
| Bread with egg, vegetable stew, and skimmed milk | Satiety & seasoning | Presentation appreciated but meal perceived as light and mildly seasoned | Modest egg portion increase; improved stew seasoning |
|  | Flavor preference | Light sweetness preferred by some | Optional light sweetening of milk |
| Soy-enriched porridge with pâté (savory fritters) | Acceptance & tolerance | Recipe positively rated for originality; soy intolerance reported by some | Provide non-soy alternative option |
|  | Portion balance | Portion perceived as slightly small | Modest porridge portion increase |
| Oat porridge with bean fritters (Ata) and skimmed milk | Texture & flavor | Porridge perceived as thick and mildly flavored | Increase liquid slightly; add light aroma or spices |
|  | Portion balance | More fritters preferred by some | Modest fritter portion increase |
| Atassi (rice and beans dish) with vegetable stew, eggs, and apple | Flavor & protein preference | Flavor perceived as mild; some preferred alternative protein options | Moderate increase in spices; optional substitution with fish or lean meat |
|  | Texture & moisture | Vegetable stew perceived as slightly dry | Adjust stew moisture moderately |
| White rice with tomato sauce and grilled fish | Flavor & aroma | Generally well accepted but perceived as mildly seasoned | Optional moderate seasoning increase; modest increase in garlic and ginger |
| Riz au gras with vegetable stew and grilled chicken | Flavor & spice level | Highly appreciated; some preferred slightly higher spice level | Optional slight spice increase |
| Macaroni with vegetable stew and grilled chicken | Spice & flavor balance | Chili level high for some; additional seasoning desired | Slight chili reduction; moderate seasoning adjustment |
|  | Accompaniment | Lower–glycemic index fruit preferred over watermelon | Substitute with lower-GI fruit option |
| Wassa Wassa (steamed cassava couscous) with vegetable stew and grilled fish | Flavor & familiarity | Generally well accepted though unfamiliar to some; stronger seasoning preferred | Moderate seasoning adjustment |
|  | Preparation preference | Cooked vegetables preferred over raw | Consider lightly sautéed vegetables |
| Akassa (fermented maize dough) with vegetable sauce and smoked fish | Taste & texture | Slight acidity and firm texture noted by some participants | Mild flavor balance adjustment; soften texture slightly |
|  | Flavor intensity | Sauce perceived as mildly seasoned | Optional slight increase in onion, chili, and salt |
| Gambali (dehulled maize flour dough) with okra sauce and grilled fish | Flavor & sauce profile | Sauce flavor perceived as mild by some | Moderate seasoning adjustment; consider flavor-enhancing ingredients |
|  | Sauce balance | Slightly more sauce preferred | Increase sauce quantity moderately |
| Telibô (yam flour dough) with tomato sauce, grilled fish, and crincrin | Flavor intensity | Flavor pleasant but mildly seasoned for some | Optional moderate increase in seasoning and spices |
| Boiled yam with vegetable stew and grilled chicken | Flavor & protein variation | Dish widely appreciated; more spice and protein variety suggested | Moderate spice increase; optional boiled egg variation |
| Beans with fried tomato and grilled mutton | Flavor & spice | Flavor positively rated; slightly stronger spice preferred | Optional slight chili increase |
|  | Portion balance | Serving size perceived as small by some | Consider modest portion adjustment |
| Vegetable salad with chicken eggs | Freshness & variety | Fresh taste appreciated; greater vegetable variety desired | Increase vegetable diversity |
|  | Condiment & serving | Condiments and bread accompaniment suggested | Optional light dressing or mustard; optional bread serving |
