# Supplementary material for: From nutritional optimization to consumer acceptance: sensory and nutritional evaluation of culturally adapted recipes for type 2 diabetes in Benin
Source: Front Nutr. 2026 Jun 26;13:1845418. doi: 10.3389/fnut.2026.1845418 (PMC13371430; doi:10.3389/fnut.2026.1845418)
Supplement: Supplementary file 3 [file Table_2.docx]

**Supplementary Table S2.** Energy and macronutrient composition of adapted recipes per 100 g as consumed (mean ± SD)*

| **N** | **Recipes** | **Energy (kcal/100 g)** | **Carbohydrate (g)** | **Protein (g)** | **Fat (g)** | **Fiber (g)** |
| --- | --- | --- | --- | --- | --- | --- |
| 1 | Bread with egg, vegetable stew, and skimmed milk | 71.1 ± 5.1 | 7.7 ± 0.6 | 4.0 ± 0.1 | 2.7 ± 0.5 | 0.4 ± 0.1 |
| 4 | Soy-enriched porridge with pâté (savory fried fritters) | 79.6 ± 3.5 | 10.0 ± 0.8 | 3.7 ± 0.2 | 2.8 ± 0.2 | 0.5 ± 0.1 |
| 2 | Oat porridge with bean fritters (Ata) and skimmed milk | 70.5 ± 1.3 | 10.7 ± 0.4 | 2.3 ± 0.1 | 2.1 ± 0.1 | 0.5 ± 0.4 |
| 3 | Atassi (rice and beans dish) with vegetable stew, eggs, and apple | 90.9 ± 5.8 | 14.2 ± 1.1 | 3.5 ± 0.2 | 2.1 ± 0.3 | 1.3 ± 0.3 |
| 6 | White rice with tomato sauce and grilled fish | 77.7 ± 3.7 | 15.5 ± 0.8 | 2.6 ± 0.1 | 0.5 ± 0.2 | 0.9 ± 0.3 |
| 13 | Riz au gras (tomato-based rice dish) with vegetable stew and grilled chicken | 97.2 ± 6.3 | 15.5 ± 0.9 | 4.1 ± 0.1 | 2.4 ± 0.5 | 0.7 ± 0.3 |
| 12 | Macaroni served with vegetable stew and grilled chicken | 80.9 ± 6.1 | 13.2 ± 1.2 | 3.7 ± 0.2 | 1.3 ± 0.4 | 0.6 ± 0.3 |
| 10 | Wassa Wassa (steamed cassava couscous) with vegetable stew and grilled fish | 119.2 ± 4.8 | 19.5 ± 0.6 | 8.1 ± 0.1 | 1.2 ± 0.4 | 1.7 ± 0.3 |
| 9 | Akassa (fermented maize dough) with vegetable sauce and smoked fish | 95.9 ± 3.3 | 6.9 ± 0.5 | 5.2 ± 0.1 | 5.5 ± 0.2 | 0.7 ± 0.1 |
| 8 | Gambali (dehulled maize flour dough) with okra sauce and grilled fish | 49.8 ± 6.4 | 7.9 ± 1.3 | 3.1 ± 0.3 | 0.6 ± 0.3 | 0.5 ± 0.2 |
| 7 | Telibô (yam flour dough) with tomato sauce, grilled fish, and crincrin (jute leaf sauce) | 63.6 ± 2.4 | 11.3 ± 0.6 | 2.5 ± 0.1 | 0.8 ± 0.1 | 0.6 ± 0.1 |
| 11 | Boiled yam with vegetable stew and grilled chicken | 81.6 ± 7.7 | 11.6 ± 0.9 | 3.4 ± 0.2 | 2.4 ± 0.8 | 0.8 ± 0.0 |
| 14 | Beans with fried tomato and grilled mutton | 157.0 ± 14.7 | 18.0 ± 1.5 | 6.4 ± 0.2 | 6.6 ± 1.2 | 1.2 ± 0.1 |
| 5 | Vegetable salad with chicken eggs | 62.4 ± 3.3 | 5.4 ± 0.4 | 3.3 ± 0.1 | 3.0 ± 0.3 | 1.0 ± 0.2 |

* Values are presented per 100 g as consumed (fresh weight basis) and expressed as mean ± standard deviation.
